# Microglial Drivers of Alzheimer's Disease Pathology: An Evolution of Diverse Participating States

**DOI:** 10.1002/prot.26723

**Published:** 2024-09-01

**Authors:** Madison K. Kuhn, Elizabeth A. Proctor

**Affiliations:** ^1^ Department of Biomedical Engineering The Pennsylvania State University University Park Pennsylvania USA; ^2^ Department of Neurosurgery Penn State College of Medicine Hershey Pennsylvania USA; ^3^ Department of Pharmacology Penn State College of Medicine Hershey Pennsylvania USA; ^4^ Center for Neural Engineering The Pennsylvania State University University Park Pennsylvania USA; ^5^ Department of Engineering Science & Mechanics The Pennsylvania State University University Park Pennsylvania USA; ^6^ Penn State Neuroscience Institute The Pennsylvania State University University Park Pennsylvania USA

**Keywords:** Alzheimer's disease, amyloid‐β, microglia, protein aggregation, proteinopathy, tau protein

## Abstract

Microglia, the resident immune‐competent cells of the brain, become dysfunctional in Alzheimer's disease (AD), and their aberrant immune responses contribute to the accumulation of pathological proteins and neuronal injury. Genetic studies implicate microglia in the development of AD, prompting interest in developing immunomodulatory therapies to prevent or ameliorate disease. However, microglia take on diverse functional states in disease, playing both protective and detrimental roles in AD, which largely overlap and may shift over the disease course, complicating the identification of effective therapeutic targets. Extensive evidence gathered using transgenic mouse models supports an active role of microglia in pathology progression, though results vary and can be contradictory between different types of models and the degree of pathology at the time of study. Here, we review microglial immune signaling and responses that contribute to the accumulation and spread of pathological proteins or directly affect neuronal health. We additionally explore the use of induced pluripotent stem cell (iPSC)‐derived models to study living human microglia and how they have contributed to our knowledge of AD and may begin to fill in the gaps left by mouse models. Ultimately, mouse and iPSC‐derived models have their own limitations, and a comprehensive understanding of microglial dysfunction in AD will only be established by an integrated view across models and an appreciation for their complementary viewpoints and limitations.

## 1 Introduction

Alzheimer's disease (AD) is a progressive neurodegenerative disorder that was first histologically characterized by neuron and synapse loss, reactive gliosis, and misfolded aggregated proteins, including extracellular amyloid beta (Aβ) plaques and intracellular neurofibrillary tangles composed of primarily hyperphosphorylated tau [1]. AD is estimated to affect 6.5 million Americans aged 65‐years and older and is projected to affect 12.7 million by 2050 as the aged population grows [2]. There are currently no proven methods to prevent, slow, or cure the disease, which in part is due to an incomplete understanding of the disease's complex etiology. Neuroinflammation, once thought to be a noncontributing consequence of AD pathology, is now recognized as an active driver of disease and may be capable of initiating neurodegeneration [3, 4]. Evidence of early immune involvement prior to the development of clinical AD [5, 6] and numerous immune‐dependent mechanisms contributing to disease pathology and neuronal injury makes regulating neuroinflammation an attractive therapeutic target [7]. In mouse models of aging and neurodegeneration, modulating immune function can prolong life and improve cognition [8, 9]. However, the shift between beneficial immune responses and detrimental neuroinflammation is difficult to distinguish, complicating the identification of successful immunomodulatory strategies [7, 10]. Neuroinflammation is largely driven by microglia, the resident immune‐competent cells of the brain [3]. In AD, microglia closely associate with amyloid plaques and neurofibrillary tangles [11, 12], and the majority of identified genetic risk factors are highly, and often exclusively, expressed by microglia, suggesting a role in disease development [4, 13].

Whether microglial dysfunction is an initiator of human AD is unknown. However, Aβ plaques and neurofibrillary tangles can exist in the brains of healthy individuals [14, 15, 16], suggesting they are insufficient to cause cognitive loss and AD. In the case of familial AD cases, although the disease presents later in life, abnormal Aβ accumulation is present since birth. Clinical manifestation may be held at bay by a homeostatic system able to compensate for accumulating pathology until mid‐life. Some have suggested neuroinflammation and microglial dysfunction, predisposed by aging, may be the determining factor in the transition from outwardly undetectable presences of pathological aggregates in the brain to cognitive loss and clinical AD [17]. This idea is supported by the existence of microglial AD risk genes and clearance mechanisms that go awry in age and with prolonged insult that will be detailed in following sections [18, 19]. Correspondingly, Aβ accumulates in the brain with age [20], after traumatic brain injury [17], and infection [21], which are all risk factors of sporadic AD. PET imaging of AD patients and healthy individuals of various ages and stages of disease reveals a synergistic association of co‐occurring Aβ, tau, and activated microglia with cognitive impairment, and its predictive ability was unmatched by co‐occurring Aβ and tau, suggesting the intersection of aberrant microglial activation and proteinopathy may be necessary for the evolution to clinical manifestations [22].

As will be explored in this review, microglia drive pathology in a variety of ways, such as in the sequestering of plaques, the hyper‐phosphorylation of tau and its spread throughout the brain, and neuronal injury by aberrant synapse engulfment and inflammatory signaling [7, 23]. Importantly, microglia‐mediated neurotoxicity may be the predominant driving force behind neuron injury and death than that caused by proteinopathy [24]. Thus, targeting microglial function may ameliorate disease by both reducing proteinopathy and neuron death. In order to successfully target microglial dysfunction in disease, however, a comprehensive understanding of their immune signaling pathways and subsequent dysregulated responses is needed. There are numerous obstacles in the way of elucidating precise targetable contributions to AD, including the largely inaccessible nature of living human microglia and the fundamental differences between human and mouse microglia, which are a more readily accessible and manipulatable option to study in widely used mouse models.

## 2 Microglial Activation States Are Context Specific

In the presence of an insult, such as misfolded protein, microglia become activated and undergo a variety of morphological and functional changes to initiate an immune response and restore brain homeostasis. The diverse responses of activated microglia depend on the identity and context of the insult, and can evolve over time. Previously, microglia were thought to adopt either a pro‐inflammatory M1 state or a neuroprotective, reparative M2 state—polarization states traditionally used for macrophages. However, recent single‐cell profiling has refuted this, with microglia often co‐expressing markers of both M1 and M2 polarization [25]. New appreciation for the heterogeneity of microglial states and their constant surveillance of the brain's microenvironment have led leaders in the field to abandon black and white incomplete classification methods, such as M1 versus M2 or “resting” versus “activated,” and call for improved classification methods [25, 26, 27, 28, 29]. Specifically, emerging classifications should include multidimensional data informed by gene expression and proteomic studies but, importantly, reflect microglial function and their dynamic nature [29].

The heterogeneity of microglial activation states in health and disease has been recognized through the single‐cell transcriptomic profiling of both human and animal microglia, demonstrating disease‐, age‐, sex‐, temporal‐, and spatial‐dependencies [30, 31, 32, 33, 34]. Microglia isolated from AD mouse models demonstrate unique gene expression signatures with worsening neurodegeneration, including the division of unique reactive phenotypes in late‐stage disease [32, 33]. In humans, single‐cell profiling of *postmortem* cortical tissue reveal multiple unique microglial signatures in both end‐stage AD patients and healthy aged individuals [35]. These results suggest that facets of microglia activity become uniquely dysregulated in neurodegenerative disease and age, though how transcriptomic signatures relate to altered function is not wholly understood and further complicated by inaccurate predictions of protein levels by gene expression [29]. Overlapping transcriptomic signatures between studies, defined by the up‐ or down‐regulation of marker genes, have been used to evaluate translational findings of mouse models and identify altered signaling pathways in disease. A widely recognized state is the disease‐associated microglial (DAM) signature [36, 37], which was first identified in AD mice. The DAM signature is characterized by a down‐regulation of homeostatic genes, including TMEM119 and P2RY12, and an increase in genes involved in lysosomal, phagocytic, and other immune response pathways and is mediated by TREM2 signaling [7, 36]. The DAM signature has been identified in numerous neurodegenerative mouse models and in both AD and healthy individuals [29], being recognized in both disease and normal aging [34]. However, the DAM phenotype is not always enriched in human microglia [38]. Other AD‐associated states distinct from the DAM phenotype have been identified through transcriptomic studies of AD mice, including those closely related to interferon signaling, with the upregulation of Oas and Ifit genes, that have been termed interferon response microglia (IRMs) [34, 39]. Interferon‐related states have also been observed in age [39]. Activated response microglia (ARMs) have been described in an AD mouse model and are characterized by increased expression of genes in MHC type II presentation, inflammatory pathways, and tissue repair; the signature also includes AD risk genes such as APOE. ARMs have overlapping features with the DAM signature and are also observed in healthy aging, but ARMs are distinct in their heterogenous subpopulations characterized by differential expression of genes involved in MHC class II presentation and tissue repair. These subpopulations are enriched in AD mice and may describe unique microglia subsets associated with amyloid plaques. However, ARMs have not yet been independently characterized by other groups or explored in other AD models. The microglial neurodegenerative phenotype (MGnD) has been described in multiple models of neurodegeneration and is defined by a shift from a homeostatic to neurodegenerative phenotype through APOE signaling initiated by a TREM2‐mediated switch [37]. Disrupting the TREM2‐APOE signaling pathway restored a homeostatic microglial signature and prevented neuronal loss in the model, translating the transcriptomic signature to microglial function [37]. Importantly, transcriptomic studies are end‐point studies and do not explicitly describe longitudinal changes of microglial activation. As such, the evolution or age‐ and disease stage‐specificity of discrete activation states have to be surmised by unique samples collected from various timepoints in pathology progression in AD animal models or from human *postmortem* tissue from individuals demonstrating varying degrees of pathology accumulation or cognitive impairment, that is, aged‐matched healthy controls, mild cognitive impairment patients, and AD patients. The latter, while using human cells, does not represent a true disease timeline, particularly in the potential of future AD development in healthy controls had death not occurred. One method to circumvent this limitation is to study human microglia in the context of predictable pathology progression in AD mice. One such study grafted human iPSC‐derived microglia precursors into the brains of AD mice, which took on disease‐relevant profiles similar to those of the endogenous mouse microglia but retained human‐specific signatures in antigen presentation, exhibited amplified pro‐inflammatory response, and demonstrated unique responses to knockout of AD risk genes [40]. However, longitudinal changes unique to human cells were not explored as this study investigated microglia states at a single timepoint.

**FIGURE 1:**
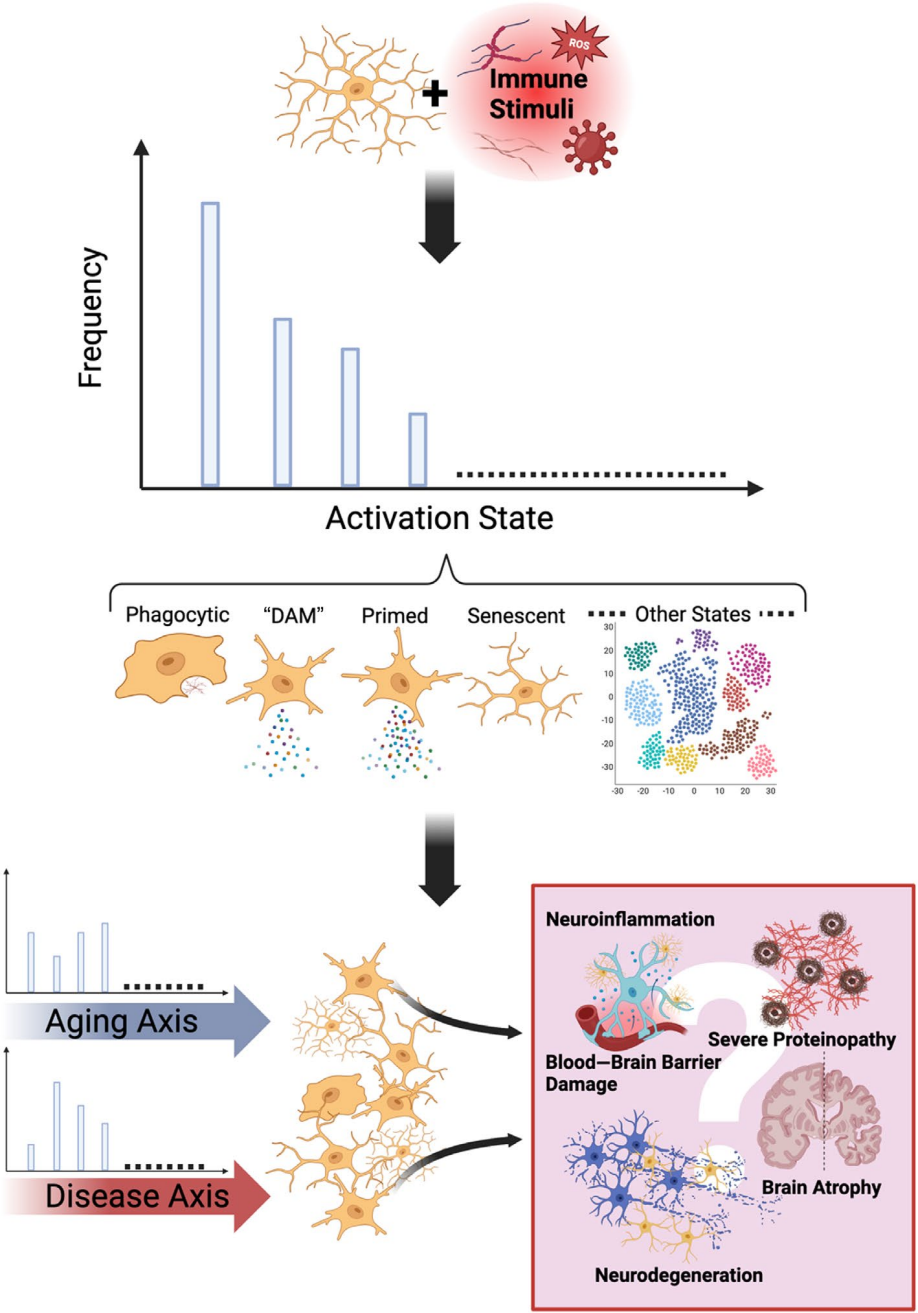
The combined actions of an evolving population of diverse microglial activation states contribute to Alzheimer's disease progression. Following an immune stimulus, microglia become activated in an effort to restore brain homeostasis. Commonly observed activation states in aging and neurodegenerative disease include primed, disease‐associated, phagocytic, and senescent microglia. Transcriptomic studies have identified a vast diversity of activation states of unknown function, and a comprehensive classification of microglial states has not been defined. Each individual state can independently contribute beneficially and/or detrimentally to AD pathology, such as the clearance or spread of misfolded proteins by phagocytic microglia or the amplified secretion of pro‐inflammatory cytokines by primed and disease‐associated microglia contributing to neuronal injury and the hyperphosphorylation of tau. The composite population of microglia, however, and its combinatorial effect of individual states drive disease. The distribution of microglial activation states and subsequent dominant downstream activities and effect on AD pathology evolves over the course of disease. Functional characterization of microglial states and their relative frequency in progressing AD is needed for the identification of successful therapeutic targets to restore beneficial microglial function or reduce detrimental dysfunction within optimal treatment windows. Created with BioRender.com.

Numerous previously reported microglial states are found in healthy aging and dissimilar diseases, such as the DAM phenotype and IRMs. The enrichment of microglial states or their unique subsets driven by specific disease context supports the idea that there is a lack of a true shift from beneficial to detrimental function and that all microglia function may be beneficial in one context but detrimental in another [29, 41]. Microglia have previously been hypothesized to have a conserved set of sensing and response functions that are utilized in neurodevelopment, healthy aging, or neurological disease in unique contexts [41]. Intermediate activation states, such as a state between a homeostatic phenotype and a DAM expression profile, have been observed [36] and trajectories of an underlying spectrum of shifting transcriptional profiles of microglia can be modeled [33], suggesting that microglial states are dynamic and regulatory elements can be leveraged to modulate the cells' function [38]. Microglial transcriptomic states represent differential gene expression and related biological pathways and may correspond to distinct immune responses or homeostatic function. Whether these responses engender a beneficial or detrimental effect likely depends on the context, such as the microenvironment, type of stimuli, duration of stimuli, age, and so on. However, the composition of the microglial population collectively drives disease, and the identification of successful therapeutic targets requires the characterization of the dominant states, their respective dysfunctions, and the synergistic/antagonist activity between them (Figure 1).

## 3 Metabolic Reprogramming Underlies Microglial Activation

Microglial activation and subsequent immune responses are dependent upon metabolic reprogramming. This relationship is termed “immunometabolism” [42]. Upon exposure to pro‐inflammatory stimuli, there is a long‐recognized shift from oxidative phosphorylation to glycolysis for microglia activation, and the balance between the utilization of oxidative phosphorylation and glycolysis regulate key microglial functions including cytokine secretion, proliferation, migration and phagocytosis [42]. While glucose is a primary fuel for microglia and is preferentially metabolized in homeostatic conditions and activation‐coupled glycolysis, ketone bodies, fatty acids, glutamine, fructose, pyruvate, and lactate are also energy substrates utilized by microglia, which make the cells highly metabolically flexible [42, 43, 44]. In the absence of glucose, microglia exhibit a decrease in oxidative phosphorylation; however, microglia proliferation, phagocytosis, and cytokine release functions persist, demonstrating microglial capacity to metabolize alternative fuels to support essential functions [45]. Correspondingly, in primary microglia and the microglial cell line BV2, when glutamine, pyruvate, lactate, or ketone bodies were the only available substrate, the cells were able to maintain oxidative metabolism, although in varying degrees [46]. Intriguingly, glutamine was the most efficient metabolite and, in its presence, there was an activation of mTOR signaling and reduction of autophagy [46], suggesting unique immune responses as a result of differential fuel utilization. Alternatively, distinct stimuli upregulate differential metabolic pathways, such as the increase in glutaminolysis, in addition to glycolysis, by IL‐1β and IFN‐γ stimulation [47]. IL‐1β and IFN‐γ stimulation was also found to suppress fatty acid oxidation and fatty acid synthesis. Pro‐inflammatory conditions exhibited increased levels of glycolytic enzymes in vivo, which was consistent with increased glycolysis observed in human iPSC‐derived microglia. In contrast, stimulation with IL‐4, traditionally regarded as an anti‐inflammatory cytokine, demonstrated increased fatty acid oxidation and synthesis with no change in other metabolic pathways [47]. Signaling pathways involved in metabolic reprogramming, specifically in the glycolytic shift, include the involvement of PFKB3, mTOR, TREM2, and HIF‐1α genes, which have all been implicated in impaired microglia metabolic reprogramming in AD [19, 48, 49]. Some risk variants of TREM2 in AD, for example, impair microglia glycolytic shift and subsequently dampens phagocytic activity [49].

Importantly, these studies demonstrate the acute reprogramming of microglia and do not inform unknown long‐term bioenergetic flexibility or metabolic reprogramming in the aging brain or neurodegenerative disease. In the AD brain, glucose hypometabolism in observed [50], and elevated glucose consumption and inflammation co‐occur in PET imaging studies [51, 52]. In addition to rapid ATP production for prompt immune responses, homeostatic microglia are motile and constantly survey the brain milieu with their processes, requiring energy‐demanding rearrangement of the cytoskeleton in their extension and contraction [44, 53]. In the diseased brain, inflammatory cells have been observed to consume as much glucose as that consumed by neurons and astrocytes in healthy brain [51]. Some have postulated that microglia may be contributing to glucose hypometabolism [43] and that microglial glucose consumption can expose other CNS cells to inadequate glucose availability for survival [51]. Given the ability of microglia to metabolize alternative bioenergetic fuels that may correspond to unique immune responses and the necessity of oxidative phosphorylation to maintain sufficient ATP production for homeostatic processes, decreased glucose availability in the AD brain may forcibly shift microglial activation states, creating a reliance on glycolysis, and impair a restoration of homeostatic function. Furthermore, negative metabolic effects of prolonged microglia activation would contribute to advancing neurodegeneration in addition to detrimental microglia immune responses, such as the accumulation of lactate from anaerobic glycolysis and its subsequent increase in acidosis, observed in the aging and AD brain, and promotion of Aβ aggregation [54]. Simultaneously, compromised immune function from inefficient ATP production by prolonged glycolytic activity would impair phagocytic activity and exacerbate the accumulation of pathological proteins [55].

How the finetuning of metabolic reprogramming corresponds to unique microglia responses or activation states is not fully understood; however, modulating immunometabolism for the dampening or enhancement of microglia immune activation is being explored for therapeutic benefit in preclinical and clinical research. The inhibition of glycolysis, for example, prevents proinflammatory activation in vitro and in vivo, defined by the reduction of proinflammatory cytokine secretion, reduction nitric oxide production, reduction of pro‐inflammatory gene expression, or a shift from activated morphology [56, 57, 58]. Masitinib, a tyrosine kinase inhibitor found to inhibit microglia and macrophage activity, is undergoing phase 3 clinical trial in mild to moderate AD [59]. NE3107 is also in phase 3 trial, thought to exert a therapeutic effect through the inhibition of inflammatory ERK‐ and NF‐κB signaling pathways [59, 60]. Increased levels of the phosphoprotein ERK has previously been found in AD mouse models and *postmortem* human AD brain tissue, and ERK was determined to be a regulator of pro‐inflammatory activation and expression of numerous AD risk genes, including Trem2, Bin1, Cd33, and Cnn2 in mice [61]. NF‐κB, a transcription factor, is a regulator of pro‐inflammatory cytokines like TNF‐α, IL‐1β, and IL‐6 [62]. GLP‐1 agonists, also being evaluated in clinical trial, have been found to produce a variety of neuroprotective effects acting on multiple cell types. In microglia, they suppress activation and the secretion of pro‐inflammatory cytokines and demonstrate protection against oxidative damage [63]. In contrast to dampening microglial proinflammatory activation, therapeutics have also been designed to drive microglial phagocytosis to alleviate the accumulation of proteinopathy. Pioglitazone and rosiglitazone, evaluated in phase 3 clinical trail are PPARγ agonists found to shift microglia to a phagocytic phenotype; however, these drugs did not demonstrate proper safety and had poor blood brain barrier penetration [64, 65]. Increasing microglia phagocytosis to reduce amyloid burden by improving aberrant gamma oscillations with noninvasive light exposure has also been explored in mouse models [66, 67, 68] and is currently being evaluated in humans in phase I/II trial (NCT03556280, NCT05637801).

An important consideration for the development of AD therapeutics is the apparent sex differences in disease pathophysiology, such as the more rapid brain atrophy and memory loss in females [69, 70, 71], and increased disease prevalence, with two times as many female patients than males [72]. Sex differences in microglia, particularly in their metabolism, have been hypothesized to contribute to increased prevalence of AD in females [73, 74, 75]. Specifically, in humans, there is sex‐specific immune signaling with decreased glutamate metabolism and increased interleukin‐10 activity in females [75]. Microglia from female AD mice exhibit increased expression of activation‐related genes and are more glycolytic and less phagocytic than microglia frome male AD mice [74, 76]. The morphology of and amyloidosis associated with the male and female AD mouse microglia corresponded with those observed of human *postmortem* tissue [74]. Importantly, it has been observed that alterations in neuroinflammatory gene expression in aging wild‐type mice occur earlier and are greater in female microglia in the hippocampus [77]. Changes in genes that are indicative of microglial activation were preferentially increased in microglia from female APP/PS1 mice, which were observed to be more glycolytic and less phagocytic and associated with increased amyloidosis. Microglia from males, on the other hand, were amoeboid, which corresponded to observations in *postmortem* tissue from AD patients. Downstream of metabolic alteration, there was also sex differences in microglial activation states, such a more rapid transition from homeostatic microglia to ARMs in female cells [34].

The diversity and dynamics of microglial responses complicate the search for specific therapeutic targets that would robustly reduce or prevent disease pathology without also disrupting beneficial activities or creating deleterious, overactive responses. Microglial function is neuroprotective by nature, acting to restore homeostasis. Dampened microglial responses, which are observed in age and after prolonged insult [16, 19, 28], may be advantageous to restore in early disease to promote beneficial function, such as phagocytosis of Aβ. However, in advanced disease, the persistent pro‐inflammatory state may promote further pathology and neuronal injury [16, 78]. As previously explored, an understanding of the disease context is necessary to determine the nature and consequence of the microglial response [41]. Given the largely unknown connection between transcriptomic states and their corresponding function, the subsequent sections will focus on microglia signaling pathways, measurable immune responses like cytokine section and phagocytic ability, and changes in proteinopathy altered in AD with the acknowledgment that these activities are governed by discrete or continuous activation states enabled by metabolic reprogramming.

## 4 Microglial Signaling Cascades Interacting With Pathological Proteins

TREM2, triggering receptor expressed on myeloid cells 2, is a transmembrane innate immune receptor found on myeloid cells. Microglia are the primary TREM2 producing cell‐type in the central nervous system [79]. TREM2 signaling modifies the activity of reactive microglia by enhancing phagocytic activity and suppressing the production and secretion of pro‐inflammatory cytokines [80, 81, 82]. TREM2 activity is often regarded as protective, as it promotes “good” debris removal activity and suppresses “bad” pro‐inflammatory functions that, although necessary for brain homeostasis, can be harmful to neurons in excess [83]. Variants of TREM2 are linked to the development of AD, and TREM2 expression is increased in the brain and CSF of AD patients and correlates with disease severity [84, 85]. It was initially thought that TREM2 activity exacerbates AD pathology [86]. However, it is now thought that increased expression of TREM2 is a compensatory mechanism in AD, where increased TREM2 signaling is a product of overcoming insufficient clearance of pathological proteins [87].

In APP23, 5xFAD, and APPPS1 models with robust amyloid pathology but an absence of tau pathology, TREM2 expression levels are increased in microglia and other myeloid cells surrounding amyloid plaques [86, 88, 89, 90]. In vitro TREM2 signaling promotes microglial clearance of Aβ [87, 88], and TREM2 knockout impairs the ability of microglia to surround amyloid plaques [86, 91, 92]. TREM2 signaling has also been investigated in the context of tauopathy. TREM2 is up‐regulated in the brains of P301S mice [93], a mouse model of primary tauopathy with neurofibrillary tangles and neuron loss present in multiple brain regions [9]. Silencing TREM2 expression in this model exacerbated tau pathology and worsened spatial learning deficits, hypothesized to be the result of increased activation of tau‐phosphorylating kinases by pro‐inflammatory factors normally suppressed by TREM2 signaling [94, 95, 96, 97]. In line with the observation that TREM2 signaling modifies the microglia response to clear Aβ and reduce neuroinflammation in amyloid models, the reduction of pro‐inflammatory factors may slow the progression of tau hyperphosphorylation by preventing the hyperactivation of tau‐phosphorylating kinases. As such, the up‐regulation of TREM2 in the AD brain may have a twofold benefit reducing tau and Aβ pathology. However, further investigation is needed to determine human‐relevant TREM2 signaling and whether the observations of TREM2 signaling are consistent in the full landscape of the disease over time. This is highlighted by inconsistent effects of TREM2 knockout over time in mouse models. TREM2 deficiency has produced both neurotoxic effects and exacerbated tau pathology in a primary tauopathy model [98, 99] as well as neuroprotection with the prevention of brain atrophy without affecting phosphorylated tau levels [100]. This could be attributed to the timeline of TREM2 knockout in the model either months before neuron loss is first observed or when substantial neuron loss is already present and suggests shifting roles of TREM2 involvement and efficacy of its modulation [7].

CX3CR1 is a microglial chemokine receptor that modulates the immune response. CX3CR1 activation regulates microglia migration and surveillance of the brain parenchyma as well as the pruning or removal of synapses and neurons during development and disease [101, 102]. In amyloid mouse models, knockdown of CX3CR1 has beneficial effects on amyloid deposition, likely as a result of increased microglial phagocytosis of Aβ [103, 104, 105, 106]. Subsequent in vitro results suggested that the reduced amyloid pathology resulted from an altered microglia activation, whereby CX3CR1 deficiency increases the phagocytic ability of microglia and modifies its cytokine secretion profile [103]. However, knockdown of CX3CR1 in mice expressing human tau resulted in increased tau hyperphosphorylation, tau aggregation, and microglial activation [107]. These results suggest that knockout of CX3CR1 has opposite effects on amyloid and tau pathology.

The classical complement cascade, part of the innate immune response, is activated in response to pathogens and apoptotic cells and recruits phagocytic cells for their clearance. In the brain, the classical complement cascade is involved in microglia's pruning of synapses—a process that is dysregulated neurodegenerative disease [108, 109]. Complement proteins are up‐regulated in neurodegenerative disease and are both expressed and detected by microglia. Histological studies have demonstrated complement activation in the AD brain, particularly in association with amyloid plaques [110]. Aβ is able to interact with the complement protein C1q to initiate the classical complement cascade [111]. Increased C1q expression and synaptic localization was observed in two AD mouse models prior to the appearance of amyloid plaques and correlated to soluble Aβ levels [112]. Inhibition of C1q, C3, or the microglial complement receptor CR3 reduced synapse loss, implicating microglial phagocytosis of synapses by the complement cascade. Additionally, microglia in wild‐type mice injected with Aβ oligomers demonstrated increased engulfing of synapses, recapitulating the phenotype observed in the amyloid mouse models. Knockout of C1q rescued this phenotype, suggesting a necessary role of complement for the Aβ‐mediated synapse loss [112]. In amyloid mouse models, although complement activation consistently exacerbates synapse loss, complement inhibition has been observed to worsen, lessen, and have no effect on amyloid pathology [113, 114, 115, 116, 117, 118]. Downstream complement protein level differences and expression of their regulatory inhibitors between mice and humans in AD pathology and age have been attributed to this discrepancy [117, 119]. In mouse models of tauopathy, complement activation is primarily linked to the exacerbation of tau pathology [116, 120].

In addition to TREM2, CD33 is another microglial surface receptor that regulates the immune response with known AD risk variants. CD33 is found to inhibit the microglial uptake and clearance of Aβ, and CD33 levels are increased in the AD brain and associated with greater cognitive decline [121, 122]. Both protective and risk variants of CD33 have been discovered [123]. Aβ accumulation is similarly affected by the risk variant PICAM. PICAM encodes phosphatidylinositol binding clathrin assembly protein which plays a role in clathrin‐mediated endocytosis and is expressed in multiple CNS cells, including microglia [123]. PICAM regulates the uptake of γ‐secretase through endocytosis and the accumulation Aβ‐42 [124]. Loss of PICAM decreases Aβ‐42 by the decreased endocytosis of γ‐secretase [124]. A protective variant of PICAM increases its expression, leading some to suggest there is protective function [123]; however, the variant's functional mechanism has not yet been explored [124]. The risk gene BIN1 encodes at least 9 isoforms of adaptor proteins in the brain and is expressed by multiple CNS cell‐types, regulating membrane dynamics and critical cellular processes [123, 125]. In AD, there is a decrease in the prevalence of neuronal isoforms and an increase of glial isoforms [126]. Its expression in neurons is thought to be protective in AD [123]. However, BIN1 expression regulates microglial proinflammatory activation [125] and its function may become deleterious in AD as a result of chronic microglial activation. Expression changes of BIN1 in microglia or dysregulated immune function as a result of identified risk variants has not yet been investigated [123]. Additionally, numerous genetic risk variants involve lipid regulation and metabolism, including APOE, PLD3, ABCA7, PLCG2, AB13, SPI1, and CLU [123]. PDL3, for one, encodes phospholipase D3 and plays a role in nucleic‐acid dependent inflammatory signaling. PDL3 expression is increased in human microglia and microglia of AD mouse models [127]. PDL3 variants have been found to double disease risk and may exacerbate the accumulation of Aβ by the dysregulation of microglial clearance mechanisms as well as altering Aβ metabolism in neurons [128].

The development of AD is influenced by numerous environmental factors, which may confer risk through “gene‐by‐environment” interactions [129] and epigenetic modifications that differentially regulate AD‐associated genes and biological pathways [130]. Clinical studies have linked obesity and its co‐morbidities, such as insulin resistance, to AD [131]. In rodent models, obesity is linked to impaired memory [132, 133], which is thought to involve exacerbated inflammation and dysregulated insulin activity by TNF‐α disruption of insulin signaling [134]. In AD patients, a high‐fat and high‐sugar diet has been linked to increased risk and earlier disease onset. A low‐sugar diet with high consumption of unsaturated fats, fiber, and protein, on the other hand, correlates to lower disease risk. However, the benefit or detriment of various diets has been shown to be patient‐specific [129]. The mechanism of obesity‐ and diet‐dependent genetic and functional changes to confer AD risk is largely unexplored, but may, in part, be driven by the gut microbiome. Nutrient composition and meal size have been observed to strongly affect the gut microbiota and influence inflammation. Overnutrition increases inflammatory tone, and, with age, synergistically acts with inflammaging, the gradual increase of persistent inflammation with age [135]. Correspondingly, diet and exercise have been shown to reduce microglial activation and neuroinflammation [73]. Obesity‐related effects also demonstrate sex differences with differential effects of high fat diet in female and male mice, with females exhibiting increased neuroinflammation, dependent on APOE genotype [136].

## 5 Both Overactive and Under‐Responsive Microglia Ineffectively Respond to and Clear Proteinopathy

Microglia become inefficient in the phagocytotic clearance of misfolded proteins with age due to a gradually increasing and persistent pro‐inflammatory state, commonly known as “inflammaging.” [16, 28, 135] In this state, microglia become “primed” in a chronic inflammatory environment, initiating exaggerated immune responses to secondary stimuli. In neurodegeneration, primed microglia more rapidly contribute to Aβ and tau pathology progression and negatively affect neuronal health and synapse plasticity [137] while simultaneously being less responsive to anti‐inflammatory cues [138]. Microglial priming and subsequent reduced clearance ability is evident in amyloidopathy models. In the APP/PS1 model, the expression of Aβ‐binding scavenger receptors and Aβ‐degrading enzymes in microglia of 14‐month‐old transgenic mice were observed to be two‐ to fivefold lower than those of non‐transgenic littermates [139]. Decreased expression of Aβ‐binding scavenger receptors and Aβ‐degrading enzymes was not observed in young transgenic animals [139]. The reduction of Aβ‐binding scavenger receptors and subsequent Aβ uptake in vitro were linked to an increase in pro‐inflammatory cytokines production [139]. Impaired Aβ clearance by primed microglia is likely influenced by both age and chronic pathological insult. In the co‐culture of 20‐month‐old APP/PS1 slices, the inability of aged microglia to clear Aβ could be reversed by the presence of young microglia from either neonatal wild‐type or neonatal APP/PS1 slices but not from aged wild‐type slices [140]. Exogenous human Aβ_42_ applied to microglia‐depleted wild‐type hippocampal slices was rapidly cleared by reintroduction of young (5 weeks) or adult (6 months) wild‐type microglia, as well as young 5xFAD microglia. Microglia isolated from aged 5xFAD cerebellum, but not forebrain, could also deplete Aβ deposits [141]. As the cerebellum in 5xFAD mice does not contain amyloid pathology [142, 143], the retained clearance capacity of both cerebellar microglia from aged 5xFAD mice and microglia from aged wild‐type mice would indicate that prior exposure to pathology reduced microglial phagocytic capacity to clear Aβ deposits, rather age or overall disease status [141]. These results would suggest that impaired microglial clearance in the presence of amyloid pathology is primarily a factor of chronic Aβ‐exposure and that naïve aged microglia can still readily engulf Aβ.

In contrast to priming, microglia can become less responsive under chronic pathological insult. In this state, microglia similarly have an impaired ability to phagocytose Aβ, linked to dysfunctional metabolic reprogramming. Exposure to Aβ induces a shift from oxidative phosphorylation to glycolysis in microglia that is necessary for an immune response [19]. Prolonged Aβ insult, however, gives rise to metabolic defects and diminished immune responses characterized by reduced cytokine secretion and phagocytosis by microglia with new immune stimuli. The microglia's “chronic tolerant phase” can be reversed by IFN‐γ stimulus to boost defective glycolytic metabolism and recover former levels of cytokine secretion and phagocytic ability [19]. Importantly, IFN‐γ treatment in 4.5‐month‐old 5xFAD mice over 3 months resulted in increased levels of phagocytosed Aβ and reduced plaque burden and neuron death [19]. Impaired metabolic reprogramming following chronic Aβ exposure may involve the mTOR‐AKT‐HIF‐1α pathway and TREM2 signaling and, in addition to IFN‐γ treatment, has been restored by cyclocreatine treatment and TRPV1 activation [48, 144]. Restoring metabolic deficits in dysfunctional microglia prevents cognitive decline in multiple amyloidopathy models [8, 19] Contrastingly, IFN‐γ signaling has been associated with worsening tau pathology and neurodegeneration through microglial‐mediated mechanisms [145].

Chronic tau exposure can similarly prevent effective microglial responses by inducing microglial senescence, which is observed in both tauopathy mouse models and human patients [146, 147, 148, 149, 150]. Microglia surrounding amyloid plaques, in contrast, are not associated with cellular senescence [148]. Cellular senescence is the permanent arrest of the cell cycle, and senescent microglia exhibit a unique secretory profile that promotes neuroinflammation. Senescent microglia exhibit impaired phagocytic abilities and motility [151], and have commonly been observed in age, stimulated by phenomena such as DNA damage and oxidative stress [152]. Tau exposure induces senescence in primary wild‐type murine microglia, which in turn leads to impaired tau clearance [146]. Removal of senescent microglia and astrocytes reduces hyperphosphorylation of tau and subsequent neurofibrillary tangle deposition and neuron death in mouse models of primary tauopathy [147, 148]. Importantly, senescent glia were removed rather than replaced, indicating that senescent glia undergo a pathological gain of function that specifically contributes to pathology progression, potentially through their senescence‐associated secretory phenotype.

The functional identifiers outlined above (primed, tolerant, senescent) describe opposing responses to chronic insults of the AD brain. In the presence of Aβ, microglia can enter a heightened pro‐inflammatory state (primed), whiles others may become less sensitive to stimuli and exhibit reduced cytokine secretion (tolerant). With tau exposure, microglia become senescent, ineffectively clearing pathological proteins while exhibiting a proinflammatory secretome, further contributing to pathology progression. Whether these states can co‐occur within brain micro‐environments, what governs the transition to (or between) each identity, and what transcriptomic signatures describe these populations has not been determined. Notably, age‐induced senescence and priming is found in normal aging but can promote neurodegeneration [153]. In aging, microglia display a diminished capacity for homeostatic function like migration and clearance [154].

## 6 Unable to Compensate, Dysfunctional Microglia Worsen Hallmark Pathology

Microglia have long been known to phagocytose and clear both soluble and fibrillar Aβ [155, 156, 157]. However, with age and chronic insult microglia lose their ability to effectively phagocytose and clear Aβ [139], and can initiate amyloid aggregation [9, 158]. It has been hypothesized that microglia may compensate for ineffective clearance by sequestering Aβ in the form of plaques to prevent neuronal damage [159, 160]. In line with this, amyloid plaque levels plateau in the brain at the onset of clinical symptoms and do not correlate with neuron death [161, 162], and extensive plaque burden can be found in the brain of cognitively healthy people [14, 163]. Aggregation of Aβ is initiated by low pH [164] and high (micromolar) concentration of Aβ monomers [165]. Low pH and high concentration of monomeric Aβ are not found in the extracellular space; thus, Aβ aggregation has been hypothesized to occur elsewhere [166]. Microglia from a combination amyloid and tau mouse model of AD (3xTg [167]) were found to have Aβ aggregates in their lysosomes [166]. Notably, while some of these microglia were associated with existing extracellular amyloid plaques, others had no existing plaques in their vicinity but contained Aβ aggregates in size comparable to small plaques. In the latter case, the lack of any nearby amyloid plaques would suggest that the lysosomal plaques did not result from internalization of existing aggregates. Non‐plaque‐associated microglia of human postmortem brains have also been found to contain Aβ aggregates, regardless of the individual's dementia‐ or disease‐status [166]. Accordingly, dense‐core plaque formation was prevented in APP/PS1 mice by eliminating receptor tyrosine kinases necessary for phagocytosis. In turn, levels of diffuse Aβ increased and amyloid deposition was observed in blood vessels, corresponding to worsened memory deficits and suggesting that plaque formation in microglial lysosomes may be protective [159]. Similarly, impaired microglial phagocytosis by TREM2 deficiency in 5xFAD mice resulted in more diffuse plaques without the presence of surrounding microglia [92]. The diffuse plaques were closely associated with increased neurite damage, which was hypothesized to be the result of exposed protofibrillar Aβ that would otherwise be contained in microglia‐sequestered dense‐core plaques [168]. These results suggest a potential role of microglia in the initial aggregation and subsequent deposition of amyloid plaques in the AD brain as a protective effort. While plaques are not regarded as overtly toxic and may shield neurons from Aβ oligomers that contribute to synapse disruption and cognitive deficits [169, 170], as a consequence of their interaction with Aβ and Aβ plaques, microglia display impaired surveillance activity and dampened homeostatic functions [171] and promote the spread of Aβ in the brain [172]. Further, chronically activated microglia can detrimentally affect neuron health in the secretion of proinflammatory factors that spur on the production of reactive oxygen species, directly injure neurons and synapses, and contribute to the accumulation of tau pathology [23]. Thus, protective efforts by microglia in the sequestering of Aβ is not a completely benign response in disease, and represents a contextual shift from beneficial function to detrimental function that is not driven by a mechanistic change.

Ineffective microglial clearance also gives rise to hallmark tau pathology. Microglia phagocytose soluble and insoluble forms of tau in vivo [173, 174, 175]. However, microglia cannot effectively break down tau and will release it [149, 173], promoting its spread in brain and likely contributing to the accumulation of tau in regions not connected by synapses [176, 177]. Human PET imaging demonstrates parallel propagation of microglial activation and tau pathology, following Braak‐like stages [22]. Microglia‐mediated tau spread has been demonstrated by the reduction of tau pathology in brain regions effected in advancing disease by the elimination of microglia in primary tauopathy models [174]. It was determined that the microglia‐mediated spread of tau was driven by the secretion of exosomes containing tau seeds (pathological tau that has the capacity to induce tau aggregation), and tau spread was similarly prevented by the inhibition of exosome synthesis [174]. Importantly, microglia from wild‐type and tauopathy mice do not express tau mRNA and, thus, do not natively contain tau; however, they contain tau seeds that are released into the culture medium in vitro [173]. Similarly, microglia cultured in medium containing tau seeds reduce but do not clear all of the tau, suggesting that microglia can uptake extracellular pathological tau but are unable to effectively break it down [173]. Microglia isolated from *postmortem* brains of AD and primary tauopathy patients also contain and release tau seeds capable of initiating aggregation [173], and microglia and various tau species co‐localize in *postmortem* brain tissue of AD patients and healthy individuals [175].

Microglia not only deposit tau seeds in pathology‐naïve regions but promote hyperphosphorylation and subsequent aggregation of tau protein. Activated microglia directly lead to tau hyperphosphorylation through the secretion of cytokines and downstream activation of a variety of kinases [94, 95, 96, 97], such as JNK/p38, GSK3β, Cdk5, and MARK [178, 179]. Microglia isolated from a combined mouse model of human tau and knockout CX3CR1, previously shown to have exacerbated tau pathology with increased microglia activation [107], are capable of inducing tau hyperphosphorylation when transferred to non‐transgenic mice [97]. Similarly, tau protein from primary wild‐type mouse neurons transfected with human tau exhibit no aggregation, but when the transfected neurons are co‐cultured with activated microglia, aggregated tau accumulates in neurites [95].

In concert with microglial spread of tau and ineffective Aβ clearance, microglial dysfunction results in a vicious circle of progressing pathology, whereby activated microglia foster a pro‐inflammatory environment, which damages neurons, hyperphosphorylates tau, and induces the production reactive oxygen species; in turn, these downstream events chronically activate the microglia and lead to undesirable, ineffective response states that further promote neuroinflammation and pathology progression. It is important to note that numerous microglial‐independent mechanisms drive disease, such as Aβ‐dependent tau phosphorylation [180, 181], prion‐like seeding, and synaptic tau spread [182, 183, 184]. Thus, restoration of beneficial microglial function may be insufficient to completely disrupt disease processes. Subsequently, effort has been made to determine the magnitude and nature of microglial contribution in the initiation and progression of disease.

## 7 Depletion of Microglia Inconsistently Ameliorates AD Protein Pathology

The depletion of microglia from the brain is a recent strategy applied to isolate the role and contribution of microglia in disease. Use of this strategy is made possible by the discovery that the reduction or complete removal of microglia in mice does not cause abnormalities in cognition or behavior [185, 186]. Widely‐used methods to reduce microglia populations are Cre recombinase strategies to ablate cells expressing microglia‐specific markers or application of inhibitors of colony‐stimulating factor receptor 1 (CSFR1) such as PLX3397 and PLX5622. CSFR1 signaling is required for microglia differentiation from yolk sac‐derived progenitors during development and mature microglia homeostasis and survival [186]. PLX5622, the more selective inhibitor, demonstrates greater than 20‐fold selectivity over homologous receptors and exhibits improved blood–brain barrier (BBB) penetrance [166, 185]. PLX5622 administration results in more rapid elimination of microglia and, unlike PLX3397, does not affect the viability of oligodendrocyte progenitor cells with prolonged treatment [187]. Both molecules have been used via cerebral injection or PLX‐treated chow to deplete or near‐eliminate microglia in the mouse brain.

The effects of microglial depletion have been studied in the context of amyloidopathy and tauopathy in mice. With existing evidence of the dual contribution of hyperphosphorylation and spread of tau by microglia, it is reasonable to expect reduced tau pathology after microglia depletion. In fact, near‐complete elimination of microglia prevents neurodegeneration and reduces the level of hyperphosphorylated tau and its spread in aged primary tauopathy mice whether treatment is initiated prior to or after the onset of tau deposition in the models [24, 145, 174]. However, without near‐complete microglial elimination, neurodegeneration still occurs, as even a small population of activated microglia can lead to neurodegeneration [24]. A moderate 30% reduction in the microglial population in aged tauopathy or 3xTg mice exhibits no effect on tau pathology, though substantial tau aggregation and neuron loss was already present at the time of treatment [185, 188]. It is not immediately clear from these studies if microglia contribute to tau pathology accumulation or neuron loss consistently over time—a crucial element of identifying therapeutic target sufficient in preventing neurodegeneration and its effective window.

In amyloidopathy models, microglial depletion largely reduces plaque burden; however, this does not always result in favorable disease outcomes. For example, while plaques are reduced in the brain tissue, deposition of Aβ in the brain vasculature is observed [166]. Interestingly, microglial depletion in amyloidopathy commonly produces cognitive improvements, which are not dependent on amyloid changes. Specifically, microglial depletion in 5xFAD mice, prior to the onset of extra‐neuronal Aβ plaque deposition, prevents considerable plaque deposition (~90%), decreases intraneuronal amyloid levels, and improves fear‐associated memory [189]. Similar reduction is found after the start of amyloid deposition [166, 190]. However, microglial depletion in later stages of the disease does not ameliorate or prevent further plaque deposition [191], although depletion prevented neuron loss, reduced overall neuroinflammation, and improved contextual memory [191]. In the 3xTg model, late‐stage depletion similarly improved cognition without modulation of amyloid or tau levels [185]. As such, microglia may contribute to neuron loss and memory impairment independently of pathology progression.

Part of the discrepancy in effect seen in microglial depletion studies is likely a result of the study's goal and experimental design; that is, some sought incomplete microglia reduction [185, 188] to determine if there was a therapeutic benefit of this approach that could be applied to human disease, while others completely ablated microglia to parse out their contribution to disease [24, 145]. A comprehensive view of the evolving timeline of microglial contribution to disease onset and progression cannot be deduced from the summation of these studies, and is particularly muddied by the distinct timeline of pathology spread in different mouse models [188]. It is worth noting that even with plaque reduction, microglia elimination did not always result in reduced levels of soluble or insoluble Aβ‐38, Aβ‐40, or Aβ‐42 [166]. This result is surprising because microglia are known to phagocytose Aβ [155, 156, 157]. Because microglia's phagocytic ability and enzymatic degradation of Aβ is impaired with age and continued inflammatory insult [139], it is feasible that the removal of already‐impaired microglia does not affect existing Aβ levels, but does impact their deposition in plaques. These seemingly contradictory results highlight the potential for specific treatment windows for modulating microglial function to achieve therapeutic benefit, but these windows would rely on the distribution of microglial states present (Figure 1) and the context in which they are found. Importantly, these studies do not explicitly explore age‐related dysfunction in the microglia. While depletion of microglia has been performed at different ages in AD mouse models, which may contribute to contrasting results such as the effect on pathology accumulation, age's unique contribution cannot be isolated from coinciding differential disease pathophysiology between timepoints. This information, however, would be critical for the understanding of age‐dependent dysregulation and microglia contributions exclusive to neurodegenerative processes.

## 8 Transgenic Overexpression AD Mouse Models Obscure the Identification of Relevant Microglia Dysfunction

Mice do not naturally develop AD, and in order to study AD pathology in mice, transgenic overexpression models of human AD mutations are used [192, 193]. Some models overexpress multiple familial mutations in the same or different genes, resulting in rapid pathology development in the animals [194, 195]. While familial AD is likely initiated by the abnormal accumulation of Aβ, as the activity of the genetic mutations would suggest [3], downstream processes also result in the accumulation of tau pathology. The co‐occurrence of Aβ and tau pathology is also observed in the sporadic form of the disease, although there is still debate over whether Aβ‐dependent mechanisms initiate sporadic cases. It is without question, however, that both characteristic proteinopathies are necessary for the current characterization of the disease, which stresses an issue with the most basic familial AD mouse models: mouse models, whether they express a singular mutation or overexpress multiple familial AD mutations, do not develop subsequent tau pathology [193, 196]. It is an egregious flaw that a model used to study disease pathophysiology does not give rise to a major disease component, and even more so that many models of this neurodegenerative disease do not experience neuron loss [192, 194, 197, 198, 199]. Some studies have introduced tau pathology by other means, such as the expression of tauopathy‐relevant mutations or injecting tau oligomers and aggregates into the brain. While artificial introduction of tau deposition by these methods may offer insight into downstream interactions caused by the co‐occurrence of Aβ and tau pathology, the natural timeline of progression and underlying mechanisms by which that pathology would arise are lost. Overexpression in itself can have extraneous effects causing disease irrelevant phenotypes [193]. Notably, familial AD is also only a small fraction of cases, estimated to account for less than 5% of all cases [200], and unknown mechanistic differences between familial and sporadic cases cannot be identified in current mouse models. The differential functions of AD risk genes have been studied in mice to gain insight into how specific alleles may increase one's risk to develop sporadic AD; however, the etiological events cannot be studied in a model that does not develop the disease.

In addition to the concerns of engendered pathology in AD mouse models, the translatability of these results is further complicated by the inherent differences between mouse and human microglia. Histologically, these differences were appreciated early on. Distinct plaque‐associated microglial activation patterns and an overall dampened immune response were observed in a familial AD mouse model compared to those of the AD brain [21, 201, 202]. More recently, transcriptomic studies have revealed an absence of numerous genes involved in the human microglial immune response in mouse microglia [203, 204], and, notably, there was limited overlap in changes in gene expression over the course of aging and neurodegeneration between human and mouse microglia [39, 203]. In aging, in particular, of the limited number of genes to be differentially expressed in both human and mouse microglia, roughly half of these genes were regulated in opposite directions [203]. The protein sequences of some microglial genes implicated as sporadic AD risk genes, including as TREM2 and CD33, are only 50% identical [205, 206]. These differences, particularly those observed in aging and neurodegeneration, are troubling for the identification of human relevant molecular targets capable of modulating the course of AD. Successful strategies in mice, including those targeting the immune response such as non‐steroidal anti‐inflammatory drugs, have not shown the same promise in humans [207]. That being said, mouse models allow for the studying of cellular functions and interactions in a whole‐body system over the animal's lifetime and can be precisely genetic modified, but there needs to be exceptional understanding for their limitations to decipher disease relevance and additional disease models and experimental methods to test the translation of findings. Induced pluripotent stem cells (iPSCs) derived from human AD patients and healthy controls, for one, offer new insights into microglial dysfunction in AD.

## 9 Human Cellular Disease Models Are Needed to Recapitulate Microglial States

The differences between murine and human microglia and the imperfections of AD mouse models stress the need for human cell models to understand the role of microglia in a disease‐relevant landscape. Especially in the case of AD where many cell types contribute to its development and progression through complex, inter‐dependent mechanisms [197], the understanding of cellular interactions in their true microenvironment is of utmost importance and is arguably the largest advantage of mouse models. However, there is a critical need for the identification and verification of human‐relevant cellular mechanisms, which in the case of microglia may largely be limited in mouse models. Advances in multi‐cell type iPSC‐derived models, highly reminiscent of the human brain in cell–cell interactions and cellular function, is making this need more achievable [206].

To bridge the gap between various mouse models and end‐point *postmortem* human tissue, which does not allow for the manipulation of living cells or reveal changes within individuals over time, iPSC‐derived models allow for the study of living human cells that cannot be isolated and cultured from mature tissues. Since their invention, numerous protocols have been developed for derivation of diverse brain cell types using two‐dimensional and three‐dimensional cell culture methods. Unlike mouse models overexpressing familial AD mutations, iPSC‐derived neuronal cells from familial patients develop both Aβ and tau pathology. Importantly, cells derived from individuals with sporadic cases of AD also develop varying degrees of pathology, creating new opportunities for studying genetic risk factors and unique mechanisms of sporadic disease [208]. iPSC‐derived models exhibit continued accumulation of Aβ and tau aggregates over months of culture [209, 210], promoting the investigation of longitudinal changes contributing to advancing disease states without the over‐expression of AD‐relevant or ‐irrelevant genes.

Numerous protocols for differentiating microglia have been developed [211, 212, 213, 214] and iPSC‐derived microglia from Alzheimer's patients and those carrying risk variants have been studied in isolation or in co‐culture with other neuronal cell types. In a comprehensive study, microglia were differentiated from iPSC lines derived from AD patients with a variety of genetic backgrounds, and isogenic controls were created to investigate how disease‐associated mutations and alleles alter microglia function and contribute to disease [215]. The study demonstrated that APOE4, the strongest and most common genetic risk factor for AD, resulted in impaired microglial phagocytic ability and mitochondrial respiration and increased cytokine secretion, while familial mutations in APP and PSEN1 did not result in robust functional changes. These results point toward a functional deficit of APOE4 microglia in response to pathological insults in AD, whereas familial mutations in APP and PSEN1 likely do not directly contribute to microglial dysfunction in disease. These findings highlight the potential of distinct microglial roles in sporadic and familial AD, and that heterogeneity in microglial dysfunction can be more readily identified using human iPSC‐derived models than in current animal models. Others have similarly demonstrated APOE4‐mediated phagocytic deficits and increased pro‐inflammatory phenotypes of sporadic AD patient‐derived microglia. Interestingly, the conversion of APOE4 to the more common APOE3 isoform ameliorated multiple disease‐relevant phenotypes, including the impaired microglial uptake of Aβ [216]. In addition to APOE, other microglia‐expressed AD‐relevant genes have been investigated in iPSC‐derived microglia, including TREM2. TREM2 missense mutations in iPSC‐derived microglia‐like cells resulted in decreased expression levels of TREM2 and subsequent impairment of migration and phagocytosis [217]. Similarly, TREM2 loss‐of‐function mutations impair the ability of microglia to shift to a glycolytic metabolism to mount an immune response and, subsequently, prevents the phagocytosis of Aβ [49].

Although iPSC‐derived cells are relatively immature as revealed by low expression of microglia‐specific genes [215, 218], studies of human iPSC‐derived microglia, complete with rigorous isogenic control strategies, will be crucial in identifying disease‐relevant pathways among complex genetic interactions. Improved iPSC‐derived microglial maturation, with gene expression and function more representative of microglia from isolated human adults, has been achieved through culture with other brain cell types [213, 218, 219, 220]. Not only do improved differentiation methods continue to overcome the shortcomings of iPSC‐derived cells for the study of adult‐onset disease, but models of increasing complexity are being developed to better reflect the cell–cell interaction and brain microenvironment, such as a iPSC‐derived tri‐culture system of neurons, astrocytes, and microglia to investigate cell‐type specific interactions in neuroinflammation [219]. Upon inflammatory stimulus, cultures containing microglia showed an increase in the complement protein C3, a common marker of neuroinflammation. C3 levels were dramatically increased in the tri‐cultures of microglia, neurons, and astrocytes as compared to those of microglia–neuron co‐cultures. It was determined that increased C3 in the tri‐culture system was induced by a microglia‐astrocyte feedback loop. The same study examined astrocyte‐microglia cross‐talk in tri‐cultures of control microglia and astrocytes with human embryonic stem cell‐derived neurons carrying a familial AD mutation or those derived from its isogenic control. C3 levels of the tri‐cultures were significantly increased in those containing familial AD neurons compared to control neurons, suggesting a neuronal and/or pathology influence in the exacerbation of astrocyte‐microglia signaling in a disease context [219].

Three‐dimensional iPSC‐derived cerebral organoids comprising various neuronal cell types allow for increased model complexity that more accurately represents native physiology with mature connectivity and cell–cell interactions. These cerebral organoids have demonstrated more advanced AD pathology compared to 2D culture methods [221, 222, 223], and will be advantageous for identifying human‐specific microglia–neuron interactions and initial screening of drugs [222, 224]. A caveat to organoid differentiation, however, is that often the inhibition of mesoderm and endoderm development is used to drive the differentiation of neuronal cells, inhibiting the development of microglia and other mesoderm‐originating cell types [218]. Recently, the development of a protocol that does not use inhibition strategies, developing self‐patterning organoids with complex cortical‐like layers and confined markers of distinct brain regions [225, 226], was found to innately give rise to microglia [218]. The organoids' microglial population, however, vary between specimens and are typically proportionally smaller than the population in the human brain. Other in vitro strategies have overcome this limitation by introducing iPSC‐derived microglia to organoids in defined quantities [216, 227], which also allow for the investigation of region‐specific microglial functional differences using modified organoid protocols that induce ventral or cortical identities [227]. This type of regional specificity is advantageous in the context of AD, where regional differences in microglial gene expression and function have been previously observed *in vivo* [31, 228] and may contribute to AD‐specific spread of pathology in the brain [229]. It is important to note that strategies incorporating microglia into already developed organoids prevent microglia from participating in early neurogenesis and network formation—key microglial roles in brain development [230]. As such, others have overcome this limitation by forming organoids with the combination of neural and macrophage progenitors whose resulting microglia contributed to synaptic pruning during neuronal network maturation [231]. Our group generated microglia‐containing cerebral organoids from iPSCs carrying a familial AD PSEN2 mutation and its isogenic control. AD organoids exhibited a unique longitudinal cytokine signature characterized by an overall reduction in cytokine secretion after an initial amplified immune response. The altered immune milieu in the AD organoids was associated with reduced synapse density, which was prevented by microglial depletion with PLX5622 treatment [232]. The AD organoids did not exhibit an accumulation of pathological proteins, which supported previous findings that the PSEN2 N141I mutation results in the dysregulated immune activation rather than altered amyloid precursor protein processing [233, 234]. Importantly, we demonstrated longitudinal changes in dysregulated immune signaling using human iPSC‐derived microglia.

These techniques can be used to identify human‐relevant cell signaling pathways, though in vitro models are not a part of a whole‐body system. Others have integrated human iPSC‐derived cells and organoids into the brains of mice to investigate them in the context of a whole organism, and in the case of organoids, introduce in vivo vascularization for improved functionalization [235, 236]. Human iPSC‐derived microglia transplanted into the brains of mice revealed that human microglia express human‐specific signatures comparable to those of the adult human brain, and that transcriptomic changes from neurological insult are species‐specific [235]. Integrating human cells in mouse models allows for the longitudinal study of human‐specific cell activity in the context of a whole‐body system. However, like mouse models, iPSC models (monoculture, co‐culture, organoids, etc.) have their own limitations [237]. While improved techniques continue to emerge, early AD organoid studies have revealed characteristic functional changes and late‐stage pathology hallmarks [210, 238, 239, 240]. Organoid models have also been used to study nongenetic factors of sporadic AD such as the potential role of pathogens in pathology development and blood–brain barrier leakage simulated by serum exposure [241, 242].

## 10 Conclusion

Microglia actively contribute to AD in a complex, multi‐faceted manner, and are likely an early driving force in disease capable of contributing to both Aβ and tau pathology accumulation and neuronal injury through diverse mechanisms. The detrimental activities of microglia are not executed by one activated population of cells, and are, instead, carried out by heterogenous populations comprising different microglial states of largely unknown origin, though influenced by the pathological, aged brain milieu. Transcriptomic studies reveal diverse sub‐groups of microglia that correlate with advancing disease and/or age, although the functional changes of each activation states have not been characterized. How the distribution of microglial activation states evolve over time, the contextual consequence of their downstream function, and their combined effect on pathology progression and neuronal injury remains unknown. Recovery of beneficial microglial function and/or the reduction of detrimental actions contributing to pathology accumulation and neuron injury would likely offer substantial therapeutic benefit. However, the presence of an evolving distribution of diverse states may mean that the modulation of one state can exacerbate the effects of another, particularly if therapies are applied at the tissue level, where microenvironments of varying levels of insults and microglial states would exist. Importantly, an understanding for whether these states are (i) static and predictable given a known microenvironment or whether (ii) microglia are able to transition between states and can be influenced to do so will inform modulation strategies. At the moment, there is a nascent understanding of the heterogeneity of microglial populations and their respective contributions to disease. There is a critical need for the in‐depth characterization of microglial dynamics over the course of the disease, not readily achievable in current AD models. The difficulty in identifying human‐relevant targets among a very complex, interwoven network of immune cues and signaling cascades is likely in part due to incomplete etiology of disease and animal‐specific differences in AD mouse models. In light of contrasting, distinct microglial dysfunctions in amyloid and tau models, future therapies should be informed by and tested in models containing both pathologies, such as human iPSC‐derived organoids which also leverage human protein–protein interactions and species‐specific signaling. However, a comprehensive understanding of microglial heterogeneity in AD will only be achieved by holistic integration of results from many types of models, with a deep understanding for their limitations.

## Author Contributions

**Madison K. Kuhn:** conceptualization, writing – original draft, visualization, writing – review and editing. **Elizabeth A. Proctor:** conceptualization, funding acquisition, writing – review and editing, supervision.

## Ethics Statement

No human subjects or animals were involved in the process of writing this review article.

## Conflicts of Interest

The authors declare no conflicts of interest.

## Peer Review

The peer review history for this article is available at https://www.webofscience.com/api/gateway/wos/peer‐review/10.1002/prot.26723.

## Data Availability

Data sharing is not applicable to this article as no new data were created or analyzed in this study.
